# Multiple blood flow measurements before and after carotid artery stenting via phase-contrast magnetic resonance imaging: An observational study

**DOI:** 10.1371/journal.pone.0195099

**Published:** 2018-04-11

**Authors:** Hisashi Tanaka, Yoshiyuki Watanabe, Hajime Nakamura, Hiroto Takahashi, Atsuko Arisawa, Takuya Fujiwara, Chisato Matsuo, Noriyuki Tomiyama

**Affiliations:** 1 Department of Radiology, Osaka University Graduate School of Medicine, Suita, Osaka, Japan; 2 Department of Neurosurgery, Osaka University Graduate School of Medicine, Suita, Osaka, Japan; Medical University Innsbruck, AUSTRIA

## Abstract

After carotid artery stenting, the procurement of information about blood flow redistribution among brain-feeding arteries and its time trend is essential to understanding a patient’s physiological background and to determine their care regimen. Cerebral blood flow has been measured twice following carotid artery stenting in few previous studies, with some discrepancies in the results. The purpose of this study was to measure cerebral blood flow at multiple time points after carotid artery stenting, and to elucidate the time trend of cerebral blood flow and redistribution among arteries. Blood flow rates in 11 subjects were measured preoperatively, at one day, one week, and about three months, respectively after carotid artery stenting by using phase-contrast magnetic resonance imaging. The target vessels were the bilateral internal carotid arteries, the basilar artery, and the bilateral middle cerebral arteries. Lumen was semi-automatically defined using an algorithm utilizing pulsatility. The results showed that blood flow rates in the stented internal carotid artery and the ipsilateral middle cerebral artery increased following carotid artery stenting. Blood flow rates in the contralateral internal carotid artery and the basilar artery gradually declined, and they were lower than the preoperative values at three months after stenting. The sum of blood flow rates of the bilateral internal carotid arteries and the basilar artery increased after carotid artery stenting, and then decreased over the next three months. There was no significant change in the blood flow rate in the contralateral middle cerebral artery. From these results, it was concluded that redistribution among the bilateral internal carotid arteries and the basilar artery occurs after carotid artery stenting, and that it takes months thereafter to reach another equilibrium.

## Introduction

Carotid artery stenting (CAS) is an effective interventional therapy for carotid artery stenosis [1–3]. The aims of CAS are to alleviate the stenosis of internal carotid arteries and to prevent artery to artery emboli [4]. In terms of regaining cerebral blood flow, studies have been conducted on blood flow changes after CAS. Through alleviating the stenosis of the carotid artery, CAS is thought to increase blood flow through the stented artery, thereby increasing total brain flow and redistributing blood flow among the bilateral internal carotid arteries and the basilar artery.

To detect its change, blood flow has been measured before and after CAS using magnetic resonance (MR) perfusion [5–8], MR phase-contrast imaging [9, 10], MR imaging with arterial spin labeling [11–13], X-ray computed tomography perfusion [14–23], nuclear studies [24–29], ultrasound [28, 30–33], digital subtraction angiography [34], and near-infrared spectroscopy [35].

Most studies measured blood flow at one time-point after CAS. The duration between CAS and flow measurement ranged from 2–3 hours [5, 26, 28] to 3–6 months [9]. Since blood flow presumably changes dynamically after CAS, measuring different time-points will be an important factor.

Some studies [7, 18, 25, 26] have measured blood flow twice after CAS. An MR perfusion study reported that although the time to peak and arrival time did not significantly change 1 day after CAS, it showed significant normalization after 30 days [7]. A positron emission tomography examination was performed before, 1 to 7 days after, and 3 to 4 months after CAS in one study [25]. It revealed that the cerebral blood flow (CBF; measured in ml/100 g/min) and cerebral metabolic rate of oxygen increased 1 to 7 days after CAS. They decreased nearly to the preoperative value at 3–4 months after CAS. Recently, single-photon emission computed tomography examination was done before, within 2 hours after, and 3 to 6 months after CAS [26]. Contrary to our expectations, CBF did not increase within 2 hours after CAS. However, CBF and cerebrovascular reactivity increased 3 to 6 months after CAS.

Unlike the above-mentioned nuclear methods, phase-contrast magnetic resonance (MR) imaging can measure the blood flow rate in individual arteries. Its use has been validated in animal studies [36, 37]. In this way, the direct effects of CAS can be measured.

Doppler ultrasound is another method to measure blood flow rate in individual arteries. Most previous Doppler ultrasound reports on CAS [28, 30–33] measured blood velocity instead of blood flow rate, i.e., volumetric blood flow, probably due to the difficulty in measuring the size of an artery. However, blood flow rate can also be measured using this method, with a precision that is similar to that of phase-contrast MR imaging [38]. Although Doppler ultrasound has advantages, like bedside availability and ease of performing multiple scans, it also has some disadvantages, including dependency on the operator’s expertise and acoustic window. In these respects, phase-contrast MR imaging is a more robust method.

Thus the aim of this study was to measure the blood flow rates of the intracranial arteries once before and thrice after CAS, and to elucidate the redistribution and the time trend of the blood flow rates after CAS, using phase-contrast MR imaging.

## Materials and methods

### Subjects

This prospective study was approved by the ethics review committee of Osaka University Hospital (approval number: 14009–3). Eleven consecutive patients who had been scheduled for carotid artery stent surgery and granted their informed consent constituted this cohort study from May 1, 2014 to April 30, 2016.

### MR study

MR examinations were performed four times, before carotid artery stent surgery (1–3 days before surgery), 1 day after surgery, 1 week (7 days) after surgery, and about 3 months after surgery. A 3T-MR system (Discovery MR750, GE healthcare, Milwaukee, WI) was used with a 32-channel head coil. Each MR examination consisted of 3D-TOF- (time of flight) MR angiography (TR 30; TE 3.4; FA 18; Matrix 512 x 256; NEX 1; ST 1.2; Slice Spacing 0.6; Slab 1; FOV 200), T2-weighted axial images (FSE TR 5800; TE 98.9; ST 5; FOV 220; Matrix 513 x 320; FA 111), T1-weighted axial images (T1-fluid attenuation inversion recovery (FLAIR); FA 111; TR 2696; TE 19; ST5; FOV220; Matrix 352 x 256), FLAIR images (FA 111; TR 8800; TE 142; ST 5; FOV 220; Matrix 320 x 224), diffusion-weighted images (FA 90; TR 7200; TE 62.9; ST 5; FOV 240; Matrix 128 x 256) and blood flow measurement.

### MR blood flow measurement

**Fig 1:**
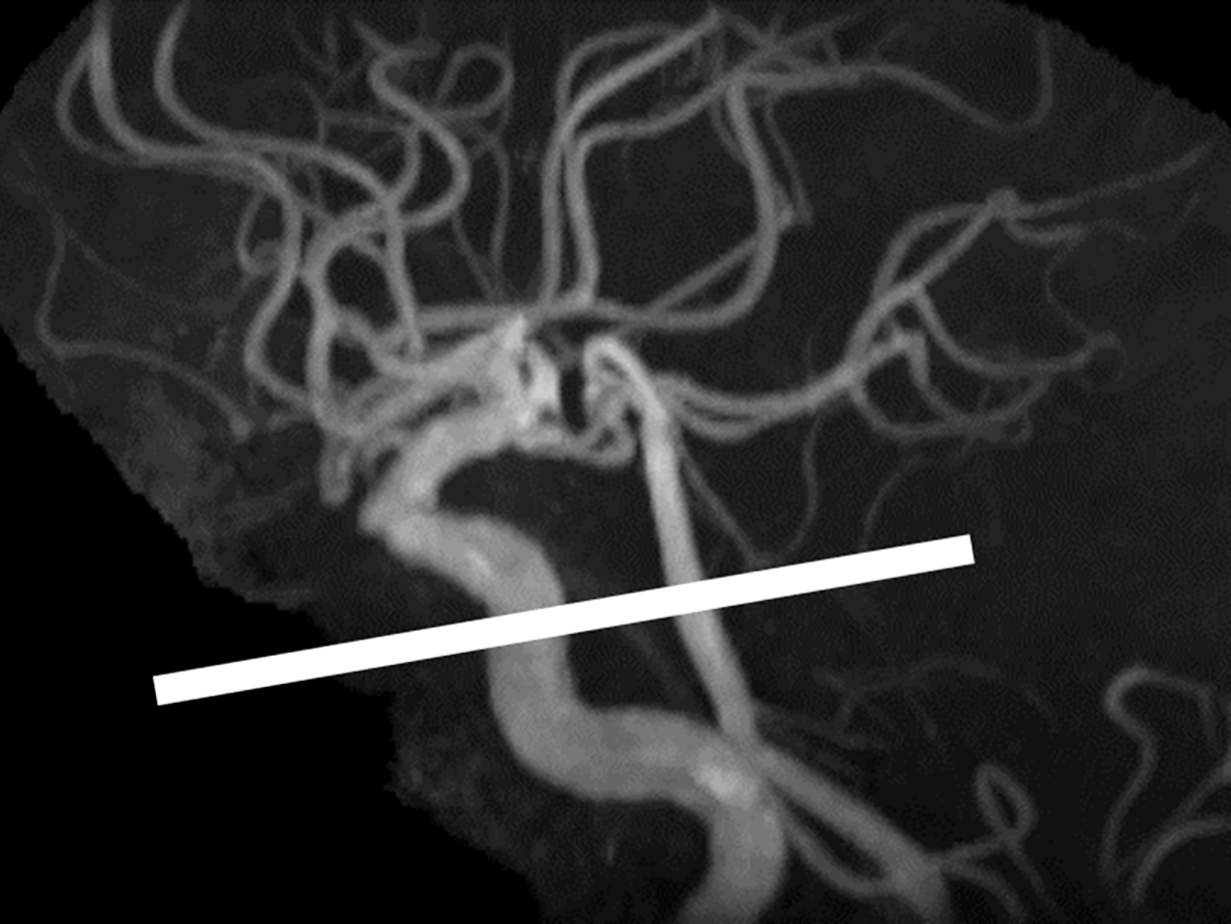
Imaging plane for blood flow rate measurement in the bilateral internal carotid arteries and the basilar artery. The plane is set as close to perpendicular as possible to these three arteries using the lateral view of the maximum intensity projection image of a three-dimensional magnetic resonance angiograph. Thus, the blood flow rates were measured in the presellar or posterior vertical segment of the cavernous portion of the internal carotid artery and basilar artery. The arteries in these anatomical locations run in a fairly straight manner and have low turbulence.

The blood flow rate was measured at the horizontal section of the bilateral middle cerebral arteries (M1) and the base of the brain (Fig 1). The measuring point of the horizontal portion of the middle cerebral artery was determined as follows. First, the straight portion of M1 was selected on MR angiography images. Second, the coordinates of the start and end points of the straight portion were measured. Third, by using the vector connecting the start and end points as a normal vector to the image plane, twelve 3D images (3D Vasc PC; TR 8.6; TE 3.1; FA 20; ST 2; FOV 120; Matrix 256 x 256; NEX 2) were obtained. Fourth, among these twelve images, one best image was selected on the basis that the section of the relevant artery was circular and no branch vessel existed. Lastly, the blood flow rate was measured at the location using fast-cine phase-contrast (TR 6.8–8.6; TE 3.5–4.4; FA 20; ST 3; FOV 120; Matrix 192 x 192; NEX 2; 16 phases; Venc 150cm). In this way, the fast-cine phase-contrast image slice is perpendicular to the relevant artery. As for the base of the brain, the blood flow was measured in three arteries: the bilateral internal carotid arteries and the basilar artery. Because there is generally no plane that is normal to all three vessels, the method employed for the middle cerebral arteries does not work. Thus, we selected twelve 3D images that were as close to perpendicular as possible to these three arteries. After acquisition of the 3D images, one best image was determined and a fast-cine image was obtained at the location (Fig 1).

### Blood flow analysis

First, MR images were examined by a radiologist to determine whether they were appropriate for further analysis. If some error was found to render the data unsuitable for further analysis, data of the corresponding vessel were excluded. Then, phase-contrast images were transferred to a PC with the MATLAB (MathWorks, Natick, MA) software installed. Blood flow was measured by one of the authors with an in-house program using the PUBS algorithm [39], which utilizes the pulsatility of arterial flow to define the lumen boundary. Using this method, an operator marks three points inside the lumen; thereafter the lumen boundary is automatically defined.

### Measurement of angles between the phase-contrast imaging slice and arteries

In order to correctly measure blood flow rate, the direction of the artery should not deviate significantly from the direction of the normal vector of the imaging slice. The phase-contrast image for the middle cerebral artery was set to be perpendicular to the middle cerebral artery, whereas the image for the bilateral internal carotid arteries and the basilar artery can be suboptimal in this respect, because only one image was obtained for three non-parallel arteries and it is impossible to set an image that is perpendicular to all three arteries. In order to evaluate this effect, deviation angles between the direction of the arteries and the normal vector of the phase-contrast image were measured. The direction of the arteries was defined as the vector connecting the points of the relevant arteries about 2.5 mm proximal and 2.5 mm distal to the image slice.

### Statistical analysis

The deviation angles were analyzed by three-way analysis of variance (ANOVA) with time of measurement, vessel, and the subject as the factors. The aim of this analysis was to determine whether the time of measurement was significantly correlated with the angles.

The flow rates of individual arteries and the sum of flow rates of the bilateral internal carotid arteries and the basilar artery were also analyzed by two-way ANOVA with time of measurement and the subject as the factors. When a variation showed a statistically significant effect according to the time of measurement, the Tukey post hoc test was used. Overall risk for type 1 errors was set at 6%. Because we analyzed with ANOVA and subsequent post hoc test six times, statistical significance was set at 1% using the Bonferroni correction for each ANOVA and post hoc test.

## Results

### Subjects

The demographics of the subjects are shown in Table 1. The subjects were 9 men and 2 women and their age ranged from 66 to 84 (mean 73 years). MR examinations were done (i) 1–3 days before CAS, (ii) 1 day after CAS, (iii) 7 days after CAS and (iv) 86–100 days after CAS. Two patients had occlusion of the internal carotid artery contralateral to the CAS side.

**Table 1:** Subjects' demographic data.

Subject/Sex/Age.y	Symptoms	ICA Stenosis, %(NASCET)	
		Ipsilateral to CAS side	Contralateral to CAS side
1/F/71	Transient ischemic attack	54	100
2/M/69	Minor stroke	74	100
3/M/68	Transient ischemic attack	50	0
4/M/72	Asymptomatic	77	65
5/M/84	Asymptomatic	77	0
6/F/71	Minor stroke	85	53
7/M/77	Minor stroke	82	27
8/M/81	Transient ischemic attack	86	0
9/M/71	Asymptomatic	74	0
10/M/66	Minor stroke	67	19
11/M/70	Asymptomatic	87	19

### Angles between the phase-contrast imaging slice and arteries

Deviation angles between the bilateral internal carotid and the basilar arteries and imaging plane ranged from 1.4 to 35.1° (mean 18.1°, standard deviation [SD] 8.3°). Three-way ANOVA revealed that the deviation angles between arteries and imaging planes were not significantly changed by the timing of MR examinations. All raw data of angles between the phase-contrast imaging slice and arteries are available as supporting information.

### Flow rates

Flow rate measurements in the bilateral middle cerebral arteries of one case (#8) at 3 months after CAS were erroneously done at different locations, probably because of mistyped coordinates. Aliasing occurred in a middle cerebral artery of another case (#9). Because of these errors, the bilateral middle cerebral arteries in the former case and the middle cerebral artery in the latter case were excluded from further analysis.

**Fig 2:**
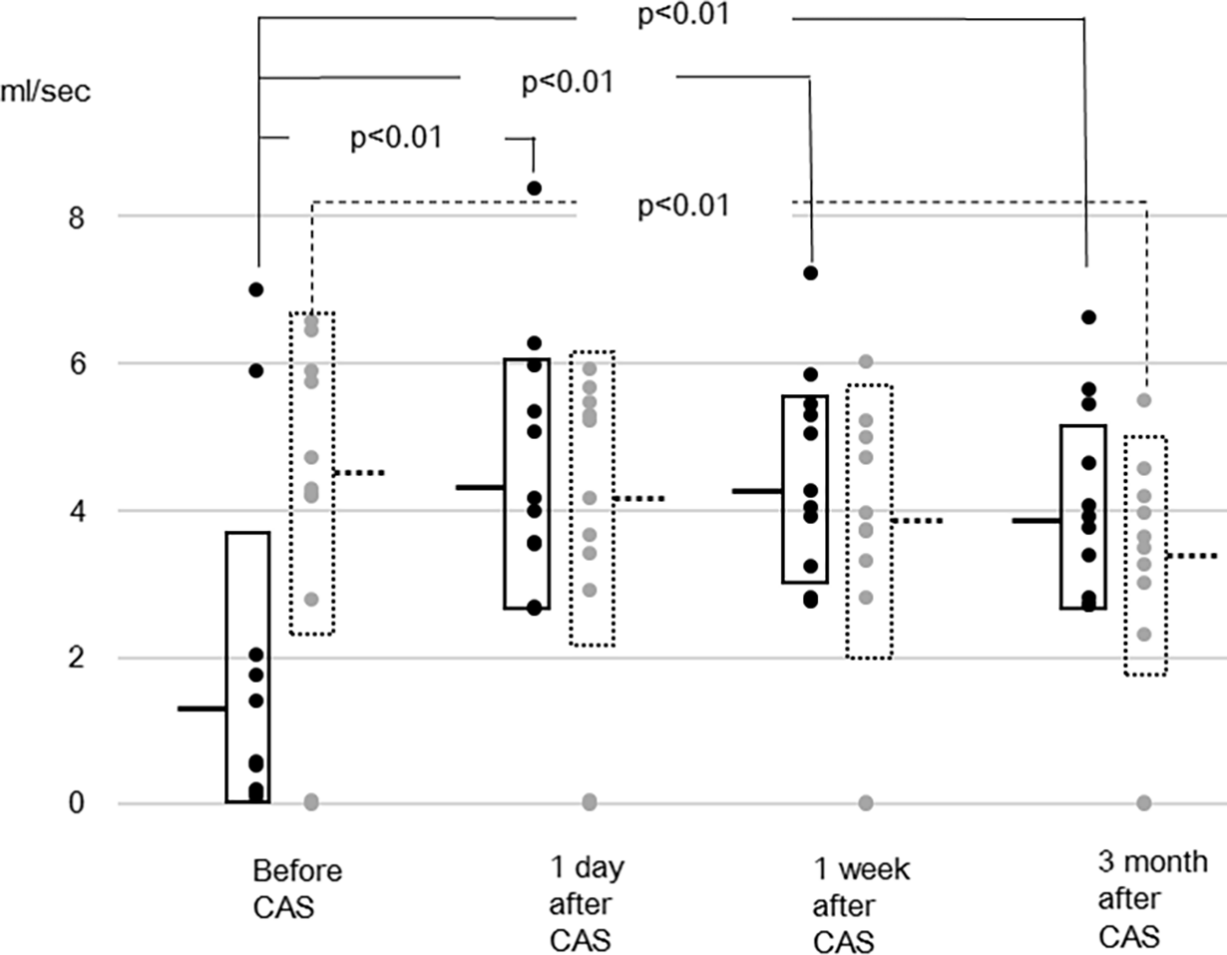
Flow rates of the stented internal carotid artery (solid line) and contralateral internal carotid artery (dotted line). Horizontal bars and boxes represent mean and mean +/- standard deviation, respectively. Compared to preoperative values, the flow rates of the stented internal carotid artery after carotid artery stenting (CAS) are significantly larger. Compared to preoperative values, the flow rate of the contralateral carotid artery at 3 months after CAS is significantly smaller.

**Fig 3:**
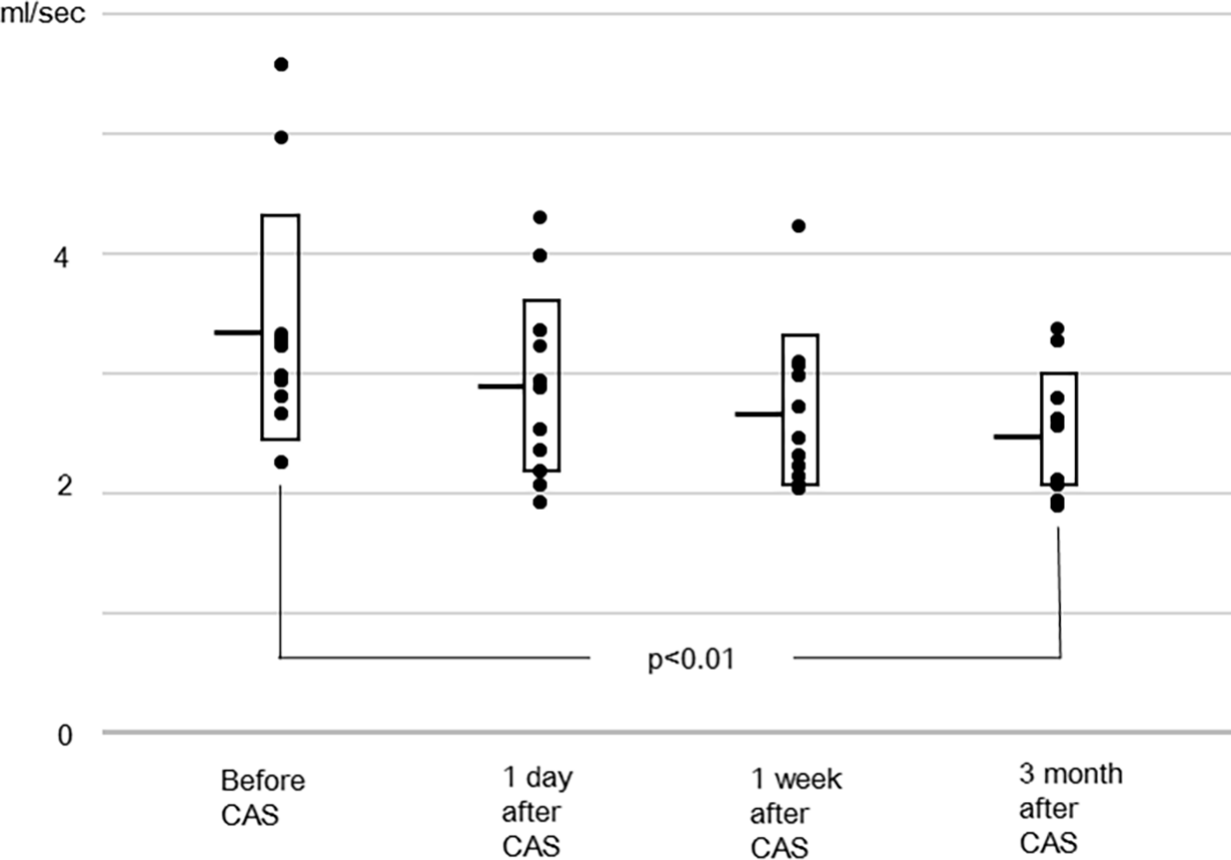
Flow rate of the basilar artery. Compared to the preoperative value, the flow rate at 3 months after CAS is significantly smaller.

**Fig 4:**
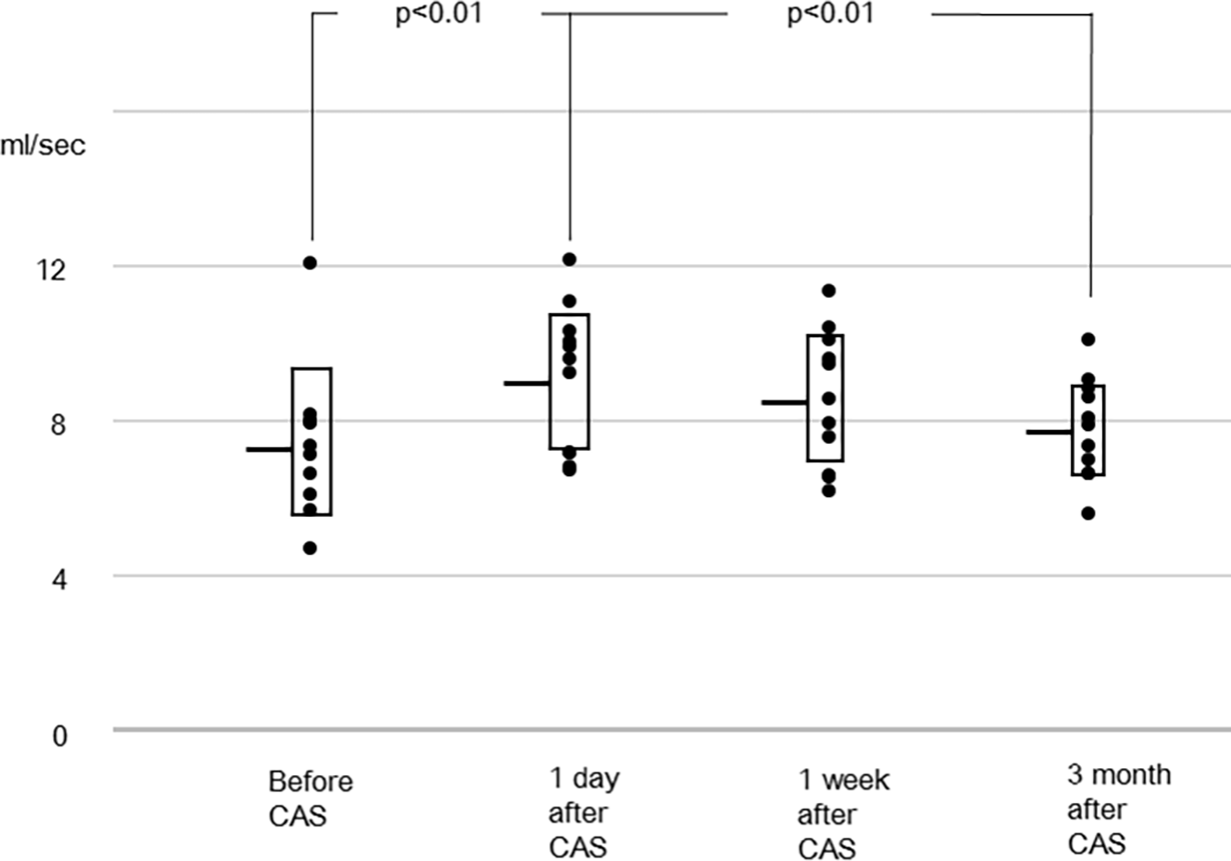
Sum of flow rates of the bilateral internal carotid arteries (ICAs) and the basilar artery. The sum increases after CAS, then decreases over the next 3 months.

**Fig 5:**
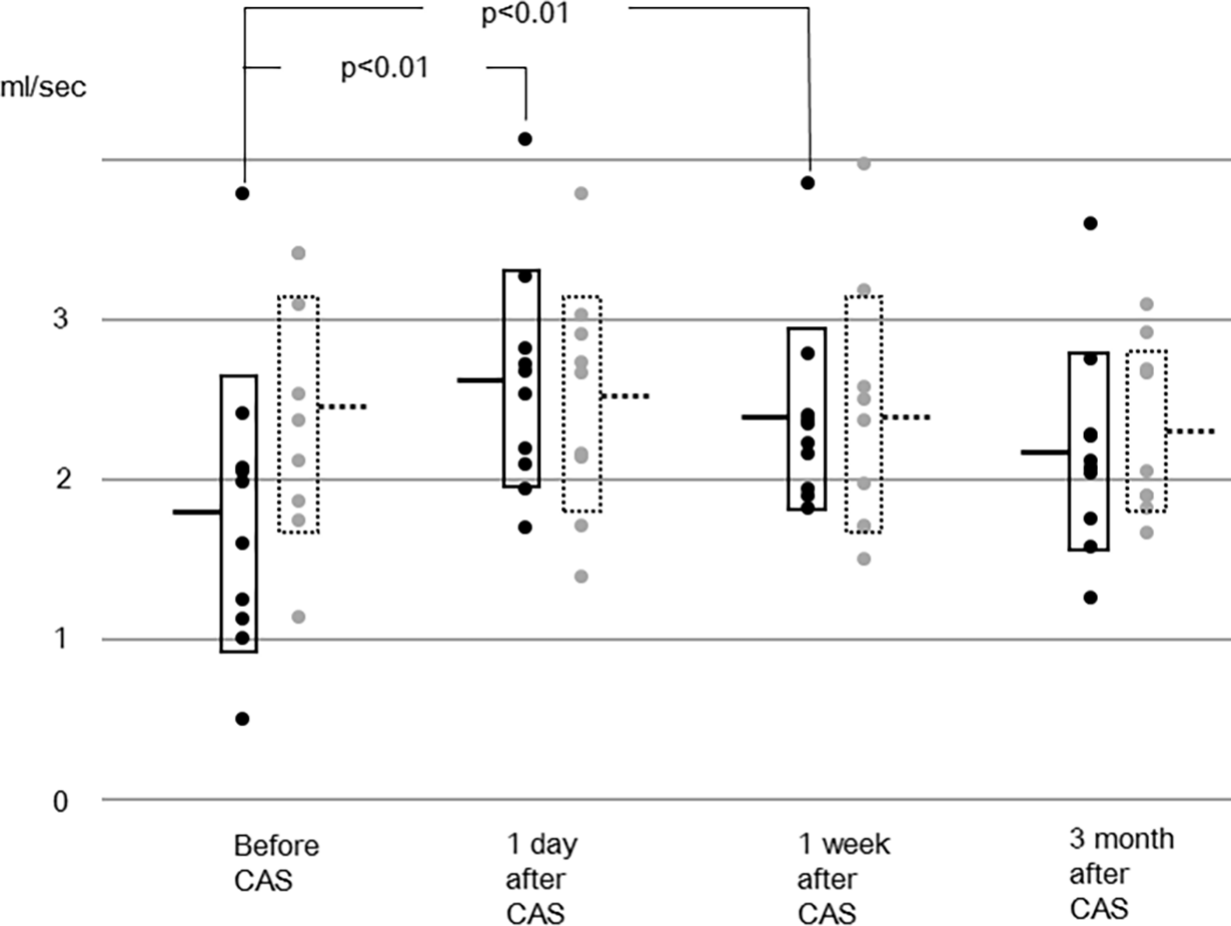
Flow rates of the middle cerebral artery ipsilateral to the stented internal carotid artery (solid line) and contralateral middle cerebral artery (dotted line). The flow rate of the stented side middle cerebral artery increases after CAS and its trend is similar to that of the stented internal carotid artery. The flow rate of the contralateral one does not show significant changes.

In the other vessels, the sum of flow rates of the bilateral internal carotid and the basilar arteries, and the flow rates of individual arteries are shown in Figs 2–5. The flow rate of the stented internal carotid artery increased after CAS (Fig 2). The flow rates of the internal carotid artery contralateral to the stented carotid artery and the basilar artery declined from preoperative values to those at 3 months after CAS (Figs 2 and 3). The sum of flow rates of the bilateral internal carotid arteries and the basilar artery increased after CAS and decreased in the next 3 months (Fig 4). The flow rate of the middle cerebral artery ipsilateral to the stented internal carotid artery was significantly larger than the preoperative value at 1 day and 1 week after CAS (Fig 5). In the flow rate of the middle cerebral artery contralateral to the stented internal carotid artery, no significant effect of time of MR examinations was found (Fig 5). All raw data of flow rates are available as supporting information.

## Discussion

In this report, CAS led to increased blood flow in the stented artery. A decrease in blood flow in the contralateral internal carotid artery and the basilar artery was observed when the value before surgery was compared with that at 3 months after CAS. The sum of flow rates of the bilateral internal carotid arteries and the basilar artery increased after CAS and showed a gradual decline over the next 3 months. The blood flow in the middle cerebral artery ipsilateral to the stented internal carotid artery increased after CAS, whereas that of the opposite side did not change.

The concomitant decrease in blood flow in the contralateral internal carotid artery and the basilar artery, although not previously described in CAS patients to the best of our knowledge, is intuitive. When an internal carotid artery territory is deprived of blood flow, the other arteries supply it via the circle of Willis or leptomeningeal anastomosis. After the stenosis of the internal carotid artery is alleviated, the contribution of collateral circulation will decrease.

The gradual decrease in the sum of blood flow rates after CAS can be explained as follows. The cerebral blood vessels are in a dilated state when a shortage of blood flow takes place. Even if the shortage is resolved by CAS, the return of the vessels to their normal state may take months. Decreased CBF was also described by a nuclear study [25] after CAS, and by a nuclear study [40] after carotid endarterectomy; these studies measured blood flow at two points postoperatively. In the first report [25], authors ascribed their findings to arteriolar self-regulation and concluded that the arterioles presumably contracted to normal size over the long term. The fact that it takes months to restore equilibrium, which signifies that no further change occurs in the time frame, may be important to understanding the underlying pathophysiologic phenomenon.

Previous studies on blood flow after CAS can be classified according to the timing of measurement after CAS. Single-photon emission computed tomography within 2 hours after CAS revealed that the CBF did not show significant changes [26]. Positron emission tomography conducted 1 day after CAS revealed that CBF increased in 16 out of 18 patients [27]. After 3–6 months, although the flow rate in stented internal carotid artery increased, that in the ipsilateral middle cerebral artery was not significantly altered on phase-contrast MR [9]. Compared to its preoperative value, CBF had increased at 3–6 months after CAS, according to one single-photon emission computed tomography study [26], but another positron emission tomography study did not show any significant differences [25]. Our results are consistent with those obtained using phase-contrast MR imaging and positron emission tomography [9, 25].

In elderly healthy volunteers (mean age 73.4 years), the rates of blood flow of the bilateral internal carotid arteries, bilateral vertebral arteries, and basilar artery were measured using phase-contrast MR [10]. Although the report did not cite the flow rates of individual arteries, it stated the sum of the flow rates of four arteries (namely the bilateral internal carotid arteries and bilateral vertebral arteries) and of three arteries (namely the bilateral internal carotid arteries and basilar artery). These were 794.10 ± 116.67 ml/min or 13.2 ± 1.9 ml/s at the C1-2 level and 861.38 ± 180.33 ml/min or 14.4 ± 3.0 ml/s at the mid-basilar level, which were larger than the values of the patients in the current study. The discrepancy can be attributed to variations in the methods, such as slice thickness (3 mm in the present study versus 5 mm), pixel size (0.63 mm x 0.63 mm versus 0.55 mm x 1.09 mm), and lumen definition (semiautomatic versus hand drawn).

Blood flow changes can be measured by many methods. Among them intravascular blood flow rate can be measured by Doppler ultrasound and MR phase-contrast imaging. The advantages of both methods are described in the introduction section. CBF can be obtained using MR perfusion, MR arterial spin labeling, X-ray computed tomography perfusion, and nuclear techniques, like positron emission tomography and single-photon emission computed tomography. Compared to intravascular flow rate, CBF more accurately reflects ischemic deficits in specific brain tissue regions. However, because of the collateral flow, effect of CAS on CBF may be modified. For example, an increase in the internal carotid artery flow rate after CAS may decrease the flow rate in the contralateral internal carotid artery or basilar artery. The increase in CBF will then not be as conspicuous as that in the intravascular flow rate of the stented artery. Since the primary aim of the current study was to elucidate the time trend of blood flow, given that the sum of blood flow rates of the bilateral internal carotid arteries and basilar artery is sufficient to meet this aim, we measured intravascular flow rate using MR phase-contrast imaging. MR examination also has several advantages over nuclear studies. It supplies information about the presence of new infarction, stenosis of arteries, and other anatomical information without radiation exposure. Blood flow measurement provides additional knowledge about hemodynamics and can be done simultaneously with other routine MR examinations.

The flow rate of the contralateral middle cerebral artery did not change after CAS in the present study. A positron emission tomography study [25], however, reported that CBF in the contralateral hemisphere increased after CAS. This discrepancy may be partly due to changes of supplying territory of the middle cerebral artery after CAS. Before CAS, because the flow rate of the middle cerebral artery ipsilateral to the stented artery is lower than that of contralateral one, the contralateral middle cerebral artery supplies the region that was originally supplied by other vessels. With alleviation of stenosis by CAS, the territory supplied by the contralateral middle cerebral artery diminishes. At the same time, CBF in the brain tissue, displayed as ml/100mg/min, increases in both hemispheres. These two effects act in opposite directions and hence the flow rate of the contralateral middle cerebral artery does not change.

Recently, the amplitudes of the standard full-field electroretinogram in asymptomatic patients were revealed to significantly increase following carotid endarterectomy in the eyes both ipsilateral and contralateral to the surgery side [41]. It can be deduced from their results and ours that after CAS or carotid endarterectomy, the flow rates in the contralateral internal cerebral artery and collateral flow to the stented side decrease, those in both the ophthalmic arteries increase, and the flow rate in the contralateral middle cerebral artery remains the same.

There are several limitations in this study. Because both internal carotid arteries and the basilar artery were measured simultaneously, the imaging planes were not perpendicular to each artery. The maximum deviation was 35.1° and, according to a previous phantom study [42], error with this deviation is within 15%. This degree of error is not serious. In addition to this, the deviation angle did not change significantly with the timing of MR examinations. Another limitation is that 3 months may not be long enough to define the time to reach equilibrium, which signifies no further change in the time frame. Since the sum of flow rates (Fig 4) at 3 months after CAS was almost the same as that before CAS and the equilibrium value should be the same or larger than the value before CAS, we assumed that equilibrium was almost reached at 3 months after CAS. This speculation was consistent with a previous report, in which CBF was measured using MR perfusion at 3 and 100 days after carotid endarterectomy [43]. In the present study, flow rates at 3 months after CAS were lower than those at 1 week after CAS. We believe that this decrease is not measurement variability, since it was observed in all measured arteries. The difference in individual arteries, however, did not reach significance. Another limitation was the absence of controls, because it was difficult to find a patient with the same degree of stenosis who did not want surgical intervention.

## Conclusion

In conclusion, flow rates of the stented internal carotid artery and ipsilateral middle cerebral artery increase after CAS. The sum of flow rates of the bilateral internal carotid and the basilar arteries, once increases after CAS, decreases over 3 months. The flow rates of the contralateral internal carotid artery and the basilar artery decrease at 3 months after CAS compared to preoperative values. It takes months to reach equilibrium after carotid artery stenting.

## Supporting information

S1 Table Flow rates and angles of each case. (XLSX)
